# Current Discoveries and Future Implications of Eating Disorders

**DOI:** 10.3390/ijerph20146325

**Published:** 2023-07-08

**Authors:** Bing Feng, Jerney Harms, Emily Chen, Peiyu Gao, Pingwen Xu, Yanlin He

**Affiliations:** 1 Pennington Biomedical Research Center, Louisiana State University, Baton Rouge, LA 70808, USA; 2 Biology Department, Centenary College of Louisiana, Shreveport, LA 71104, USA; 3 The Division of Endocrinology, Diabetes and Metabolism, Department of Medicine, The University of Illinois at Chicago, Chicago, IL 60612, USA

**Keywords:** eating disorder, binge eating, anorexia nervosa, bulimia nervosa

## Abstract

Eating disorders (EDs) are characterized by severe disturbances in eating behaviors and can sometimes be fatal. Eating disorders are also associated with distressing thoughts and emotions. They can be severe conditions affecting physical, psychological, and social functions. Preoccupation with food, body weight, and shape may also play an important role in the regulation of eating disorders. Common eating disorders have three major types: anorexia nervosa (AN), bulimia nervosa (BN), and binge eating disorder (BED). In some cases, EDs can have serious consequences for an individual’s physical and mental health. These disorders often develop during adolescence or early adulthood and affect both males and females, although they are more commonly diagnosed in young adult females. Treatment for EDs typically involves a combination of therapy, nutrition counseling, and medical care. In this narrative review, the authors summarized what is known of EDs and discussed the future directions that may be worth exploring in this emerging area.

## 1. Introduction

**Figure 1:**
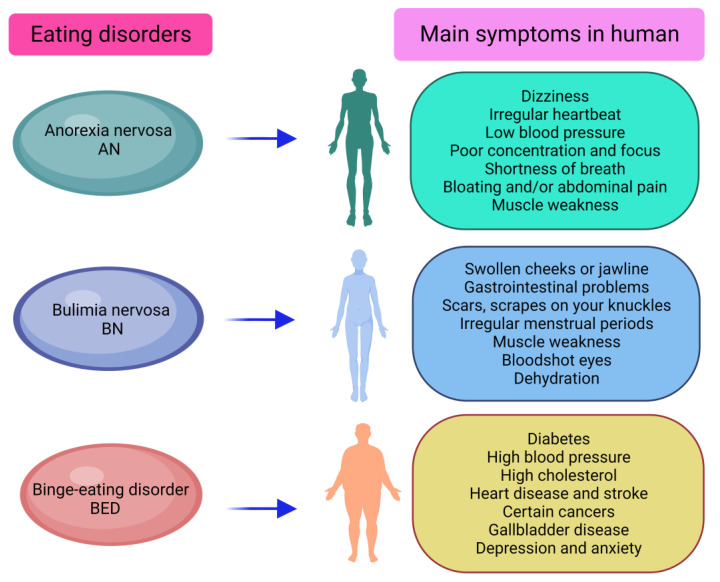
Three major eating disorders and their symptoms in human subjects (AN: anorexia nervosa; BN: bulimia nervosa; BED: binge eating disorder).

Eating disorders (EDs) are serious mental illnesses marked by dysfunctional eating behaviors and distorted body image [1]. Seven different eating disorders are identified in the list of international disease classification systems. These EDs include the well-known anorexia nervosa (AN), bulimia nervosa (BN), binge eating disorder (BED) (Figure 1), and three other disorders: rumination disorder, pica, and avoidant food intake disorder. Some otherwise specified feeding or eating disorders are listed in another category [2]. Worldwide, the three most frequent eating disorders in humans are AN, BN, and BED [3,4]. AN is defined as disturbing experiences of weight or size, inappropriate influence of size and weight on self-assessment, or persistent lack of awareness of the seriousness of current low body weight [3]. BN is defined by repeated and inappropriate compensatory behaviors to avoid weight gain [5]. BED is delineated by eating more food in short time periods than most people would eat in similar time periods and in similar circumstances [3,6]. Most people affected by these eating disorders are young women in Western countries, suggesting that the existence of sex-related problems in the etiology of EDs is irrefutable [7]. The studies have shown that, although data regarding the incidence of EDs on the community level are limited, the broad occurrence of AN among women might be 1–4%, and 1–2% for BN and BED [1].

More than fifty years of records on EDs show that less than fifty percent of patients experience complete remission, about thirty percent experience residual symptoms, and twenty percent develop chronic disease [8]. Additionally, pressure from families and medical staff further exacerbates concerns about body image and food [9]. The Lancet Psychiatry Committee’s recent synthesis report stated that people with mental disorders will have significantly higher chances of developing obesity, diabetes, and metabolic syndrome and close to two times increased risk of metabolic disease compared with individuals not diagnosed with mental disorders [10]. Eating disorders are complex disorders with increasing prevalence and are a physical and mental health concern [11]. Many genetic, environmental, and social factors lead to the development of eating disorders. Biological risk factors for eating disorders include genetic factors and the psychological element of an eating disorder that coincides with a diagnosis of another disorder. Environmental factors include the dynamics surrounding the individual, which may consist of family dynamics.

This review aims to summarize and discuss the current discoveries and future direction of three different eating disorders, AN, BN, and BED. By gathering and analyzing the most recent research and clinical evidence, this review can provide a comprehensive overview of the current state of understanding on eating disorders. It will increase public awareness and understanding of the importance of etiology and management of eating disorders. Clinicians and researchers can use the information provided in this narrative review to improve their understanding of eating disorders and develop more effective treatment strategies. In conclusion, this review can be an important tool for clinicians, researchers, and the general public in improving their understanding and treatment of these complex eating disorders.

## 2. Methods

A rapid scoping review focused on eating disorders was conducted. The definition adopted here was to map the existing literature to determine the volume and coverage of the topic, ascertain the types of literature available, and identify the gaps in the current eating disorder research. The authors searched PubMed and PsycINFO databases using keywords such as “eating disorders,” “anorexia nervosa,” “bulimia nervosa,” “binge eating disorder,” and “treatments” and included studies published in English between 2000 and 2023 that focused on eating disorders. The authors present the findings on eating disorders in tables, figures, and narrative summaries. This narrative review was reported following the PRISMA Scoping Reviews’ (PRISMA-ScR) Checklist.

## 3. Current Discoveries of Eating Disorders

### 3.1. Anorexia Nervosa (AN)

AN is an eating disorder that is characterized by strict restrictions of food intake, leading to abnormally low body weight and an intense fear of gaining weight [12]. While past epidemiological studies have focused on young women in Western countries, anorexia nervosa has been reported in men and women of all ages worldwide. This eating disorder may increase the risk of death fivefold or more [5]. AN is seen as a result of its psychological profile: the drive toward thinness and physical dissatisfaction. AN shows significant heritability as family studies have shown a substantial prevalence of AN in the first-degree relatives of a proband, who are about 11 times more likely to develop AN than controls [13]. Many concepts have been suggested by different scientists to explain and understand how AN is developed and maintained [14].

AN results in a notable reduction in bone mineral density (BMD) in approximately 40% of patients and a three times increased lifetime fracture risk [12]. Another noticeable skeletal phenotype concerning anorexia is the increased number and size of bone marrow adipocytes despite a lipodystrophic response. Clinically, there is a general negative correlation between BMD and bone marrow adipose tissue (BMAT). Unique fat depots are of particular interest in AN study [12]. Bone health in adolescents with AN has been extensively reviewed [15]. It is reported that non-stress fractures are more common in women with AN and stress fractures are more common in oligomenorrhea athletes with AN [16].

AN is an eating disorder characterized by chronic energy deprivation leading to suppression of the hypothalamic–pituitary–gonadal (HPG) axis owing to decreased gonadotropin-releasing hormone (GnRH) secretion, as observed in functional hypothalamic amenorrhea (FHA) [13]. Common variants of neurotrophic signaling genes, including brain-derived neurotrophic factor (BDNF), NTRK2, and NTRK3, appear to lead to receptivity to eating disorders [17]. Given the close association between FHA and AN and the evidence that food restriction is one of the major risk factors for the development of FHA, it can be hypothesized that genetic variants involved in satiety, appetite, and weight regulation may be responsible for the physical and/or psychological effects, as well as the differential response to stressors and the consequent suppression of the FPG axis [13].

AN is associated with neuroendocrine and immune system abnormalities. For example, leptin, an essential appetite modulator involved in AN development, also regulates immune responses through microglia-induced inflammation by increasing the expression of tumor necrosis factor-α (TNF-α) and interleukin-1b (IL-1b) [18]. Additionally, activity-based anorexia (ABA), an AN model in rodents, reduces cortical astrocyte density, suggesting that astrocyte loss and subsequent neuroinflammation may be involved in the neurobiology of anorexia [19,20]. Astrocytes may participate in eating disorders through their regulation of neurotransmitters such as dopamine and serotonin, which are known to play a role in the regulation of appetite and food intake. Astrocytes can regulate these neurotransmitters by taking up dopamine or serotonin and releasing it if necessary. Astrocytes can also modulate the activity of the dopamine neurons or serotonin neurons that produce them. Conversely, restoring glia-mediated neuroinflammation has been shown to increase glutamate transporter expression and ameliorate diet-induced anorexia (DIA) and ABA vulnerability in rodents [21]. Based on evidence from mouse models of anorexia nervosa, glial cells have been suggested as potential therapeutic targets for AN [19,20,22]. Some other therapeutic targets that focused on neurons were also reported. A recent study that focused on dopamine (DA) and serotonin (5-HT) neurons revealed that feeding behavior is regulated by intensity-dependent interactions between 5-HT neurons and DA, which could contribute to the pathophysiology of AN [23]. Hickey et. al. reported that agouti-related peptide (AgRP) releasing neuron activity in ABA animals showed a poor inhibitory response to food, independent of changes in basal activity [24]. AN may also be conceptualized as a form of genetic resistance to hunger, thus allowing for a particularly strong personal commitment to perpetuate food restriction [25]. High circulating leptin concentrations indicate that a positive metabolic balance associated with endocrine changes significantly and rapidly improves several symptoms of anorexia, including increased body agitation and the impulse to move. The proposed hypothesis is testable and could be incorporated into the design of future double-blind placebo-controlled studies using leptin as a treatment strategy in AN [26,27].

Both anxiety and depressive disorders may predispose human subjects to AN according to epidemiological data and genome-wide association studies. Furthermore, genome-wide association studies have shown that metabolic factors play a key role in AN [14]. Based on current knowledge, it is reasonable to hypothesize that the gut microbiota–brain (GMB) axis disturbance is the core pathology of AN, and that alterations in the gut microbiota caused by malnutrition could be an early step in the pathogenesis of AN. Understanding the effectiveness of interventions designed to restore normal regulation of eating behaviors in patients with AN is critical [14].

In conclusion, AN has been an eating disorder epidemic in adolescent and adult women worldwide for decades. AN requires more surveys and scholarly investigations to allow us a more comprehensive understanding. Overall, there is still much we do not understand about AN, and further research is required to improve our understanding of the disorder and to develop more effective treatments. Future studies could also investigate other neural mechanisms that will explain more unknowns in the field of AN.

### 3.2. Bulimia Nervosa (BN)

In 1979, bulimia nervosa (BN) was first described by British psychiatrist Gerald Russell as a chronic phase of anorexia nervosa. BN is characterized by binge eating followed by compensatory mechanisms including self-induced vomiting, prolonged starvation, laxatives, etc. It was reported that the estimated lifetime prevalence of BN ranges between about 0.3% and 1.6% according to the studies conducted in the USA [28]. A systematic review reported a lifetime prevalence of 0.8% for BN. Females and young adults are more prevalent in all eating disorders, with a female-to-male ratio between 3:1 and 8:1 [29,30].

BN is characterized by occasional binge eating and purging behavior. Neural processes of self-regulation, gustatory reward, and body image are thought to be implicated in the pathogenesis of BN. The study by Wang et al. showed that patients with BN exhibited abnormal increases in multiple left nodes in the mesolimbic reward circuit, lateral temporo-occipital cortex, precuneus, and right nodes in the dorsolateral prefrontal cortex [31]. A decrease in global efficiency was observed in cortical limbic circuits and somatosensory and visuospatial systems. Several corticolimbic nodules were significantly associated with BN symptoms. Their study showed BN-related changes in prefrontal control, mesolimbic reward, somatosensory structures, and white matter connectivity in the visuospatial system [32]. The aversive interoceptive experiences may be specifically associated with the pathophysiology of BN because it is well documented that there is link between avoidance of unpleasant physical sensations and BN symptoms. During aversive homeostatic perturbations, excessive anticipatory responses and abnormally reduced responses have been shown to promote hallmark binge eating behaviors such as overeating, dietary restriction, and purging [33]. Several studies give support for homeostatic instability in BN, and these altered brain activation patterns may become new targets for pharmacology, neuromodulators, and behavioral interventions [33].

When subjected to food stimulation, BN patients display less inhibitory control. Binge eating can also be used for the diagnosis of BN in clinical studies. Thus, some appetite-related hormones that regulate feeding behavior have also been extensively studied [34]. It has been suggested that abnormal ghrelin responses to satiety may lead to binge eating behaviors in patients with BN. Another hormone called leptin is produced by fat cells. Leptin suppresses appetite and causes satiety. Many findings have shown that leptin levels are significantly lower in BN patients than in healthy controls [35,36]. In the acute fasting and refeeding trials, plasma leptin levels were consistently significantly lower in BN patients than in healthy controls. Notably, fasting-induced reduction in circulating leptin is also significantly different, with 58% in healthy control women and only 7% in women with BN. These studies suggest that ghrelin and leptin are important in the development of BN, opening the door to further understanding of how appetite-related hormones interact with BN [37]. Besides ghrelin and leptin, asprosin, a novel orexigenic adipokine, may also contribute to the development of BN. Hu et al. first reported plasma asprosin concentrations in BN patients, and their study showed that overeating and uncontrolled eating are associated with increasing asprosin concentrations. In addition, increases in asprosin levels and increased depression may explain the increased frequency of loss of control [37].

Further, some additional references have reported that a neurotransmitter can be used in the treatment of BN. Mihov et al. presented the first evidence for increased metabotropic glutamate receptor subtype 5 (mGlu5), which plays a vital role in addiction distribution-to-volume ratio (DVR) in BN [38]. Their findings suggested that drugs that inhibit mGlu5 may have therapeutic potential for BN.

Subcortical shape abnormalities were examined in a large sample of adolescent and adult BN patients by Berner et al.’s study [33]. Their findings suggested a link between morphological changes in the basal ganglia structure and BN symptoms. This precise localization of subcortical morphometric changes may ultimately aid in the identification of risk markers and BN persistence, which is the first step in determining the trajectory of BN or specific structural markers for maintenance from adolescence to adulthood [39,40].

BN is a severe mental illness with prospectively dangerous complications. Family relationships play a critical role in the development or persistence of the condition. To gain a better grasp of the role of food in family interactions among BN adolescents, Lecomte et al. studied the dynamics of their families in contrast to those of adolescents with anorexia nervosa. It confirmed the need for a systemic approach beyond individual therapy and the benefits of establishing family interactions that do not involve food to rebuild communication, including those with siblings [41].

It appears that changes in the microbiota modulate appetite regulation in patients with BN. Unlike AN, BN is noticeably lacking in data. Although BN is also a life-threatening disease, studies have assessed the part of the microbiome in BN that focused only on the bacterial ClpB protein in patients and did not investigate differences in gut microbiota from healthy control groups. Like AN, patients had higher plasma ClpB levels than in healthy control groups. E. coli-produced ClpB can mimic α-MSH and stimulate autoimmune responses. BN differs from AN in the switching of IgG autoantibody epitopes that form immune complexes in BN patients [28,42].

Data proves that, except for BN, the prevalence of food addiction (FA) is higher in binge eating disorders than in other EDs. People affected by eating disorders were more likely to be addicted than those with bulimia, even though the results were not statistically significant. BN, FA, and BED have overlapping symptoms that may lead to these outcomes. Several studies support the description of BN as an addiction-like eating behavior, reinforcing the addictive nature of the disorder [43,44,45].

In summary, BN is a severe, potentially life-threatening eating disorder with less known pathophysiology mechanisms. BN is hard to beat but effective treatment can help individuals eat healthier, reverse serious complications, and feel better. More deep research is needed to improve our understanding of BN and to develop more effective treatments for the BN disorder.

### 3.3. Binge Eating Disorder (BED)

BED is described as the intake of lots of food in a short time, typically with a preference for highly palatable foods, affecting about 5% of American adults [46]. It is unclear what causes binge eating in humans, and there are limited effective treatments for BED patients. In the development of BED in humans, impaired brain serotonin (5-HT) signaling has been observed. For example, increased brain 5-HT re-uptake was found in binge eating patients, thus lowering 5-HT content [47]. In addition, the role of the 5-HT precursor L-tryptophan is significantly diminished in binge eating patients, possibly because of dysfunction of the 5-HT receptor and/or tryptophan hydroxylase-2 (TPH2), the enzyme that synthesizes 5-HT in the brain [48].

Binge eating is an important public health problem because of its close relation to other medical and psychiatric disorders, especially obesity and depression. Developing more effective binge eating treatments is an urgent need [46]. The neuronal matrix of binge eating can sometimes contribute to obesity, but the mechanism is not yet known. Zhang et. al. discovered [49] that optogenetic activation of immediate overeating was caused by γ-aminobutyric acid (GABA) neurons or their axonal projections to paraventricular thalamic (PVT) excitatory neurons in the amorphous zone (ZI) of mice [50]. Minimal intermittent stimulation of ZI GABA neurons resulted in weight gain, while ablation of these neurons reduced weight. Additionally, stimulation of excitatory axons from the hypothalamic nucleus to the paraventricular thalamic neurons or direct stimulation of paraventricular thalamic glutamate neurons reduced food intake. Their data suggest that ZI GABA neurons have unexpected orexigenic potential [49,51].

As the most diagnosed eating disorder, BED affects more than three times the number of women than men. A gut-secreted hunger hormone called ghrelin is altered in BED. Prins et al.’s research showed that ghrelin deficiency affects behavior and metabolism in binge eating mice [52]. Ghrelin deficiency does not affect the development of BED; it will alter the timing of food intake, motor activity, and metabolism. However, the interaction of ghrelin and BED in different sexes has not been fully elucidated [52]. When overweight human subjects were compared to normal weight subjects during and after the Trier Social Stress Test (TSST), Micioni Di Bonaventura et al. found BED patients had increased ghrelin levels [53]. The ghrelin system and ghrelin O-acyltransferase (GOAT) enzymes are increasingly complex regulations involving food regulation, and hunger stimuli, food choice, and obesity may play a key role in bulimia, triggering reinforcement mechanisms associated with food rewards and impulsive behavior [53,54].

Dopamine is another peptide that may be involved in BED. Yu et al. reviewed some important parameters of dopamine in BED animals and humans including dopamine receptor availability/affinity, dopamine activity, and dopamine modulator levels/activity [55]. While most studies supported these changes, the direction of the change is unclear. In future studies, it will be beneficial to carefully control for confounding variables. More importantly, some vertical studies are needed to test if a transition from a dopaminergic to a hypodopaminergic state occurs during BED development. If possible, researchers could test whether genotype modulates the relationship between BED and dopamine [55].

Binge eating episodes, characterized by uncontrollable and painful consumption of large amounts of palatable food, are the cardinal features of binge eating disorder. The inflammatory markers are altered in discrete brain regions that could contribute to food intake. Alboni et al. found that binge-like eating significantly downregulated the IL-18 or receptor system, as reflected by increased expression of the pro-inflammatory cytokine IL18 inhibitor and reduced expression of the IL-18 binding chain 18 receptors, and increased the expression of iNOS by threefold, particularly in the anterior tuberculous region of the animals’ hypothalamus [56]. Their data suggest the therapeutic potential to centrally target selected markers of inflammation to prevent the development of eating disorders.

BED is also very common in obesity and type 2 diabetes (T2DM) patients and is often associated with higher BMI [57]. Studies have shown that up to 20% of people with T2DM have an underlying BED. The prevalence of BED appears to be much higher in patients with T2DM than the prevalence of 2% to 3.5% in the general population [58]. Researchers have found medications that significantly decrease the frequency of binge eating and overweight. Abbott et al. demonstrated that a significant proportion of adults with pre-existing T2DM have clinical BED [57]. More studies are needed in exploring the importance of BED in the treatment of T2DM and development of long-term diabetic complications. If health providers are able to screen and diagnose eating disorders in human subjects in the early stage, they could provide novel antidiabetic therapies for them [57].

Recent randomized controlled findings have suggested improvements not only in overeating and purging episodes related to the diagnostic dimensions implicated in BED but also in food-related emotional responses in terms of anxiety and food cravings [59,60]. Although BED is common and limited treatment options exist, recurrence rates after treatment are often high. However, the neurobiological mechanism of BED is still less understood [50]. Hildebrandt and Ahmari examined preclinical approaches by breaking them down into two clinically significant bulk food components: short-term food consumption and loss of dietary control [50]. They suggested that the guidelines identify the most common and effective methods for modeling components of BED behavior using preclinical methods. How current preclinical models can be combined with techniques using targeted neurobiological approaches are discussed in their work. They suggested that proposed ways are needed to improve the translation of preclinical work into human studies of BED to enhance understanding of BED behavior [50,61].

Obesity is a major global public health problem, and its prevalence has been steadily increasing over the past few decades. The World Health Organization reported that more than 1.9 billion adults worldwide are overweight, and more than 650 million of them are obese. In addition, an estimated 41 million children under the age of five are overweight or obese. It was reported that obesity is a major risk factor for premature mortality [62,63]. In addition, obesity is associated with a higher risk of developing several chronic conditions that can lead to premature death, such as cardiovascular disease, type II diabetes, and some cancers [64,65]. Based on the scientific evidence presented so far, tailored comprehensive multidisciplinary treatment is essential to provide adequate care for patients with obesity and BED by addressing their overweight and its consequences. This interdisciplinary approach should combine a structured lifestyle treatment plan with healthy meal planning, PA, and behavioral interventions, according to a multidisciplinary team of experts [66,67]. They suggested that measurements for multidisciplinary interventions of adolescents with BED should consider weight loss as well as behavioral and mental improvements. More research should focus on the physical and mental effects of different forms of exercise [68,69]. Binge eating may be a behavior designed to reduce chronic stress resulting from repetitive negative thinking focused on eating or not dependent on eating. Although clinical models may partially support these hypotheses, further research is needed to directly test these hypotheses [70,71,72].

Lisdexamfetamine dimesylate (LDX) is currently the only drug approved by the FDA for the treatment of BED [73]. Still, little is known about the behavioral mechanisms underpinning the effect of LDX in the treatment of BED. Schneider et al. were the first to document the behavioral and neurological characteristics of the effects of a dose of 50 mg Lisdexamfetamine dimesylate in binge eating women [74]. They found that LDX has multiple effects on enhancing satiety, reducing responses related to food rewards, and improving cognitive control. Their results underscored the potential effect of the thalamus in mediating LDX to reduce appetite. This was achieved by altering the balance of extrinsic and interoceptive control. These data support the conclusion that new drugs (such as LDX) used for BED treatment may be most effective if their effects on satiety, reward, and cognitive processes are combined [74,75]. Based on the current range of studies, new drugs to treat BED are expeditiously needed. Heal et al. discussed the similarities in the psychopathology of attention deficit hyperactivity disorder (ADHD) and BED, and the pharmacology of drugs that have been shown to be effective in the treatment of both disorders [76]. Drugs that enhance noradrenergic and dopaminergic neurotransmission and/or are effective in ADHD are the most promising areas for new treatments for BED, analysis suggests. The lipid-derived messenger oleoylethanolamide (OEA) acts through central noradrenergic and oxytocinergic neurons to signal satiety that inhibits food intake. A recent study investigated the anti-binge effects of OEA in a rat model of binge-like eating. The results showed that systemically administered OEA prevents overeating in a dose-dependent manner [77]. They provided evidence that OEA may be a new potential pharmacological target for the treatment of binge eating behavior.

To summarize, BED is a severe mental illness worldwide. It is the most common eating disorder in the USA and can have serious physical and mental consequences. Risk factors for developing BED include a history of dieting, body dissatisfaction, and a family history of eating disorders or other psychological health conditions. Antidepressant medications may help reduce binge eating behaviors in some people with BED. However, more research is needed to develop more effective treatments for BED with fewer side effects.

### 3.4. Treatments of Eating Disorders

Over the past 20 years, the eating disorders field has made significant progress in successfully translating basic eating disorders risk factor research into preventive interventions with significant potential to reduce its risk factors, symptoms, and future ED episodes. Efficacy has been documented in multiple randomized controlled trials [78,79]. Treatment for eating disorders often involves a combination of psychotherapy, medication, and nutritional counseling (Table 1). Specifically, cognitive–behavioral therapy is a common form of psychotherapy that can help individuals with eating disorders change their negative thought patterns and behaviors related to food and body image. Antidepressant and antipsychotic medications may also be prescribed to help manage the emotional and psychological symptoms of the disorder.

## 4. Discussion

### 4.1. Limitations of the Current Eating Disorders Research

In recent decades, there has been significant progress achieved in eating disorder research through both basic and clinical research. On the one hand, several animal models have been developed for AN and BED. Research has also shown that there are biological factors that contribute to the development of eating disorders. For instance, researchers have found that individuals with anorexia nervosa have alterations in brain structure and function, as well as abnormal levels of certain hormones and neurotransmitters. More and more studies are focusing on prevention strategies for eating disorders. Body-positive curricula are used for promoting a positive body image and preventing the development of eating disorders. On the other hand, ongoing research is looking for more effective treatments for eating disorders in humans. In addition to CBT, other treatments such as family-based therapy and interpersonal psychotherapy have been shown to be effective for certain types of eating disorders. More importantly, new treatments, such as virtual reality therapy and medication have also been introduced for eating disorder treatment.

However, there are also some limitations to eating disorder research. For example, there is no good animal model for AN, BN, or BED eating disorders in basic science research. One reason could be that eating disorders are complex disorders that involve a merger of biological, psychological, and social factors. Animal models are often limited in their ability to capture the complexity of human behavior and psychology. Additionally, animal models may not fully replicate the environmental and social factors that contribute to the development of eating disorders in humans. Unlike human patients, animals cannot report their own symptoms, which makes it difficult to determine whether an animal is experiencing an eating disorder. Furthermore, the definition and classification of eating disorders in humans have evolved over time, making it difficult to establish a standardized set of criteria for an animal model. In clinical research, the limitations include small sample sizes, self-report biases, recruitment biases, and lack of diversity. More importantly, the underlying mechanisms of eating disorders are still unknown. This gap makes it very hard to move clinical research forward in a short time. While significant progress has been made in understanding the neurobiological, psychological, and environmental factors that contribute to eating disorders, there is still not much we know. Further research is needed to better understand the underlying mechanisms of eating disorders and to develop more effective treatments.

### 4.2. Future Direction of Eating Disorder Research

Considering the refractory nature of ED and the lack of evidence-based treatment, there is an urgent need to identify new approaches. The noncompetitive N-methyl-D-aspartate receptor antagonist ketamine, recently approved for treatment-resistant depression, has rapid and potent antidepressant effects [3]. The evidence presented by Ragnhildstveit et al. provides a conceptual but succinct summary of the use of ketamine for the treatment of ED [8]. Ketamine may provide the greatest utility in clinically unresponsive individuals who are resistant to the psychological, dietary, and pharmacological interventions used in standard practice and are prone to long-term ED disorders. Further studies are needed to explore the efficacy of ketamine on ED and its psychopathology, especially in terms of different subgroups, types of diagnostic dependency severity, and lifespan. These data can then be used to build safety profiles, optimize dosing, and provide targeted treatment strategies at the individual patient level [3].

More research is needed to discover the genetic component of eating disorders, as it is genetically linked to immune system dysfunction, which can have serious repercussions. Certain groups of people appear to be particularly susceptible to eating disorders; however, all people affected by this disorder experience severe physical manifestations. Although numerous studies have been conducted to investigate the adverse effects of eating disorders, more studies are needed to better understand the long-term effects and address the appropriate treatment of eating disorders [121]. Future research could focus on identifying specific genetic factors affecting the development of eating disorders, as well as exploring the interaction between genetics and environmental factors.

The COVID-19 pandemic, which is associated with social restrictions, has profoundly affected people’s psychological health. It can be hypothesized that the symptomatic behavior and psychological health of eating disorder patients deteriorated during this period. Future longitudinal studies are needed to determine whether increased depression or anxiety contributes to ED symptoms, or whether patients with existing mental illness use unhealthy eating behaviors as a response mechanism in the absence of a sense of control. This question has important implications for understanding how severe psychosocial stress affects ED symptoms in the entire population. An understanding of the mechanisms associated with dietary habits will guide us to make more targeted and empowered public health decisions to manage ED. In addition, methods to improve online therapy in the future should also be introduced [122]. The study by Gorrell et al. provides an overview of research focused on teletherapy in EDs, with a particular focus on how the COVID-19 pandemic has affected interest and job demand in the field [123]. Overall, larger randomized designs are needed.

Last but not least, future research could focus on developing and testing new treatments, including pharmacological, behavioral, and psychosocial interventions. It is critical to develop more new treatments for eating disorders, as most of the current treatments are not always effective for every patient. For example, researchers may develop some new medications that modulate the levels of neurotransmitters such as serotonin or dopamine. Researchers could also investigate the potential use of medications that regulate hormones such as ghrelin or leptin, which are involved in appetite regulation. More importantly, newer forms of CBT, such as enhanced CBT (ECBT), may be more effective than traditional CBT for some individuals with eating disorders.

## 5. Conclusions

This narrative review aims to summarize the current discoveries and progress on three different eating disorders: AN, BN, and BED. Current research on eating disorders has uncovered many important findings, including the causes, risk factors, and effective treatments for these complex mental health conditions. For example, eating disorders have multiple causes, including biological factors such as genetics and brain chemistry, mental factors such as body image and self-esteem, and social factors, for instance, societal pressure to lose weight and cultural messages about body shape and size. Early intervention and treatment for eating disorders will achieve better outcomes and reduce the risk of chronic health problems and relapse. Proper medications (e.g., antidepressants, antipsychotics, and mood stabilizers) can be helpful for the treatment of BN and BED.

Recent research on eating disorders has highlighted the complexity of these conditions including the significance of early period intervention measures in treatment, the role of genetics and social factors, and the impact of the COVID-19 pandemic. Eating disorders are associated with a range of negative health outcomes, including malnutrition, heart problems, and gastrointestinal issues. Eating disorder prevalence is increasing worldwide, with more individuals being diagnosed each year. It is important for people with eating disorders to seek professional help and for society to continue to raise awareness and provide support for those affected by these conditions.

In conclusion, eating disorders are severe mental conditions that can have serious impacts on an individual’s physical and mental health. The current discoveries on eating disorders suggest that these are complicated psychological health conditions that are influenced by a range of genetic, environmental, and psychosocial factors. Published references indicate that eating disorders affect people of all ages, genders, and cultural backgrounds. Although the exact cause of these disorders is not completely understood, a combination of genetic, environmental, and psychological factors is thought to play a role. There are several effective treatments for eating disorders, including psychotherapy, medication, and nutrition counseling, but not all individuals respond equally well to these interventions. Early intervention is key in treating eating disorders, as the longer an individual goes without treatment, the more difficult it can be to achieve full recovery.

Overall, current discoveries on eating disorders suggest the need for continued research into the underlying causes, risk factors, and effective treatments for these complex mental health conditions. With continued research, it is hoped that individuals with eating disorders will receive the support and care they need to achieve a full recovery.

## Figures and Tables

**Table 1:** Treatments of different eating disorders.

Eating Disorder	Treatments (Non-Drug)	Medications
Anorexia nervosa (AN)	Short-term hospitalization [80,81,82].	There is no single effective drug approved for the treatment of anorexia nervosa.
	Home-based therapy is the evidence-based treatment for teenagers with anorexia [83,84].	Anti-anxiety medications help anorectics to comply with nutritional rehabilitation programs [85,86].
	Nutritional counseling: Nutritional counseling is the main line of nutritional recovery and is designed to teach anorexics about their body’s need for food and essential nutrients [87,88,89].	Antipsychotics are also recommended in selected AN patients, who are not to eat in spite of ongoing anti-anxiety medications [90,91].
	Individual therapy: Anticipatory behavior therapy, specifically augmentative cognitive–behavioral therapy, contributes to adults [92,93].	Altered regulation of the hormone leptin may play role in the persistence of anorexia nervosa [94,95].
Bulimia nervosa (BN)	Psychotherapy: Also called talk therapy, this type of counseling can include cognitive–behavioral therapy, family-based therapy, and interpersonal psychotherapy [96,97,98].	Antidepressants, such as selective serotonin re-uptake inhibitors (SSRIs) (including Celexa, Lexapro, Prozac, and Zoloft) in combination with psychological therapies, are now a mainstay in bulimia therapy [99,100,101].
	Dietitian support and nutritional education: A nutritionist can design a meal plan to help develop healthy eating habits 102,103].	No medications are approved to treat bulimia nervosa.
Binge eating disorder (BED)	Cognitive–behavioral therapy (CBT): CBT is highly effective in reducing the number of binge eating episodes in individuals [104,105,106].	Lisdexamfetamine dimesylate (LDX) is currently the only drug approved by the FDA for the treatment of binge eating disorder [73,74,107].
	Interpersonal psychotherapy: It can help reduce binge eating triggered by bad communication abilities and relationships [108,109,110].	Weight loss drugs: Xenical, Contrave, Qsymia, etc. [111,112,113].
	The act of dialectic behavior therapy: It reduces the desire to overeat through studying behavioral skills that can help regulate emotions and perfect relationships with other people [114,115,116].	Topiramate (Topamax), an anticonvulsant antidepressant [117,118,119,120].

## Data Availability

Not applicable.
